# Inference of Gene Regulatory Networks Incorporating Multi-Source Biological Knowledge via a State Space Model with *L*1 Regularization

**DOI:** 10.1371/journal.pone.0105942

**Published:** 2014-08-27

**Authors:** Takanori Hasegawa, Rui Yamaguchi, Masao Nagasaki, Satoru Miyano, Seiya Imoto

**Affiliations:** 1 Bioinformatics Center, Institute for Chemical Research, Kyoto University, Uji, Kyoto, Japan; 2 Human Genome Center, The Institute of Medical Science, The University of Tokyo, Minato-ku, Tokyo, Japan; 3 Department of Integrative Genomics, Tohoku Medical Megabank Organization, Tohoku University, Sendai, Miyagi, Japan; Queen's University Belfast, United Kingdom

## Abstract

Comprehensive understanding of gene regulatory networks (GRNs) is a major challenge in the field of systems biology. Currently, there are two main approaches in GRN analysis using time-course observation data, namely an ordinary differential equation (ODE)-based approach and a statistical model-based approach. The ODE-based approach can generate complex dynamics of GRNs according to biologically validated nonlinear models. However, it cannot be applied to ten or more genes to simultaneously estimate system dynamics and regulatory relationships due to the computational difficulties. The statistical model-based approach uses highly abstract models to simply describe biological systems and to infer relationships among several hundreds of genes from the data. However, the high abstraction generates false regulations that are not permitted biologically. Thus, when dealing with several tens of genes of which the relationships are partially known, a method that can infer regulatory relationships based on a model with low abstraction and that can emulate the dynamics of ODE-based models while incorporating prior knowledge is urgently required. To accomplish this, we propose a method for inference of GRNs using a state space representation of a vector auto-regressive (VAR) model with *L*1 regularization. This method can estimate the dynamic behavior of genes based on linear time-series modeling constructed from an ODE-based model and can infer the regulatory structure among several tens of genes maximizing prediction ability for the observational data. Furthermore, the method is capable of incorporating various types of existing biological knowledge, *e.g.*, drug kinetics and literature-recorded pathways. The effectiveness of the proposed method is shown through a comparison of simulation studies with several previous methods. For an application example, we evaluated mRNA expression profiles over time upon corticosteroid stimulation in rats, thus incorporating corticosteroid kinetics/dynamics, literature-recorded pathways and transcription factor (TF) information.

## Introduction

Transcriptional regulation, which is controlled by several factors, plays essential roles to sustain complex biological systems in cells. Thus, identifying the structure and dynamics of such regulation can facilitate recognition of and control over systems for many practical purposes, *e.g.*, treatment of diseases. To accomplish this, many mathematical methods have been developed for the analysis of high-throughput biological data, *e.g.*, time-course microarray data [1]–[3]. In addition, recent technological advances have facilitated experimental discoveries, *e.g.*, DNA-protein interactions and the pharmacogenomics of chemical compounds. These contributions have allowed the knowledge of GRNs to accumulate.

For elucidation of GRN dynamics, time-course observational data have been generally used. Currently, one strategy to elucidate transcriptional regulation using observational data is to apply an ordinary differential equation (ODE)-based approach, which can represent the dynamic behavior of biomolecular reactions based on biologically reliable models, *e.g.*, the Michaelis-Menten equation [4] or the S-system [5], which are described by differential equations. Thus, this approach can recapitulate the complex dynamic behavior of biological systems [6], [7]. In this approach, several methods have been proposed to infer regulatory structures [8], [9], to reproduce the dynamic behavior of biological systems recorded in the literature [10]–[13] and also to improve literature-recorded pathways so as to be consistent with the data [14]. However, nonlinearity of the system results in an analytically intractable problem of estimating the parameter values that minimize loss function with updating simulated results. Thus, under this statistically efficient paradigm [15], this approach cannot be applied to ten or more genes to infer regulatory structures if the missing information is extensive [10].

In contrast, a statistical model-based approach using highly abstracted models, *e.g.*, Bayesian networks [16]–[18] and the state space model [19]–[22], has been successfully applied to infer the structure of transcriptional regulation from biological observational data. Because these methods simply describe biological systems, hundreds of genes can be handled computationally with ease. Whereas methods relying purely on data need to consider all possibilities of transcriptional regulation, some studies have further incorporated other information, *e.g.*, protein-protein interaction networks (PINs), literature-recorded pathways and transcription factor information [23]–[27]. Although these methods can infer relationships among hundreds of genes simultaneously, high levels of abstraction can also generate false regulations that are difficult to interpret biologically. Thus, when several tens of genes are handled with partially understood relationships, highly abstract models can be insufficient to represent biological systems. In this case, there is an urgent need for a method that can infer system dynamics and the structure of GRNs based on a model with a low abstraction that can emulate the dynamics of ODE-based gene regulatory models incorporating existing biological knowledge.

We propose a novel method for inference of GRNs based on a newly developed model that uses a state space representation of a vector auto-regressive model (VAR-SSM) [21], [28], [29]. The model is a type of state space models constructed from a typical gene regulatory system that is described by differential equations within a linear Gaussian model. The method can infer the dynamic behavior of gene expression profiles and the regulatory structure for several tens of genes by assimilating time-course observational data. Furthermore, the method is capable of integrating the existing biological knowledge, *e.g.*, literature-recorded pathways and intracellular kinetics/dynamics of chemical compounds, and can deal with even non-equally spaced time-course observational data. A regulatory structure is inferred by maximization of the *L*1 regularized likelihood. To this end, we developed a new algorithm to obtain active sets of parameters and estimate a maximizer of the *L*1 regularized likelihood using the EM algorithm.

To demonstrate its effectiveness, we compared this method to a state space model (SSM) [21], a general VAR model using LARS-LASSO algorithm [30], GeneNet [31], [32] based on an empirical graphical Gaussian model (GGM), dynamic Bayesian networks using first order conditional dependencies [33], GLASSO [34] based on sparse GGM and the mutual information-based network inference algorithms: ARACNE [3], CLR [35] and MRNET [36] by implementing artificial simulation models. The first two observational datasets are generated by two simulation models representing pharmacogenomic pathways [37], [38], including drug kinetics/dynamics, described by difference and differential equations, respectively. These pathways are initiated by the drug stimulation and observational data are obtained as non-equally spaced time-course data. The next observational dataset is generated by GeneNetWaver [39], [40] using a yeast network that is a part of DREAM4 (Dialogue for Reverse Engineering Assessments and Methods) challenge. As an application example, we applied the proposed method to corticosteroid pharmacogenomics in rat skeletal muscle [37], [38], [41]. Because this system has been investigated previously through biological experiments, corticosteroid kinetics/dynamics and the related genes are already partly elucidated. Therefore, we incorporated time-course mRNA expression data (observational data), candidate genes/pathways related to corticosteroids, intracellular corticosteroid kinetics/dynamics and, additionally, TF information from ITFP (Integrated Transcription Factor Platform) [42]. As in the simulation experiment, the observational data were obtained as non-equally spaced time-course data (GSE490) after stimulating rat skeletal muscle with corticosteroid. Consequently, we propose candidate pathways for extensions of corticosteroid-related pathways and their simulation dynamics in the presence of corticosteroid.

## Methods

### Linear Description of Biological Systems from ODE-based Models

For gene regulatory systems, we postulate a general hill function-based model of transcriptional control, in which each gene has a synthesis process (regulated by other factors) and a degradation process, described by a differential equation [43], [44]. Let 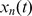 be a time-dependent function representing the abundance of the *n*th (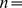 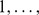 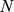) mRNA in a cell, where 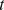 means time. Further, we consider subsets of 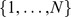, 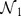 and 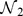 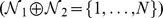, whose regulatory functions are described by two different forms [38], [45], [46]. Then, the time-evolution of 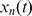 is represented by
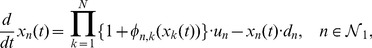
(1)

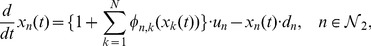
(2)where 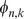 represents the regulatory effect of the *k*th gene on the *n*th gene as a hill-function, 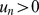 and 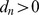 are the synthesis and degradation rates of the mRNA, respectively. For example, in a previous pharmacogenomic study [38], 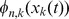 was represented by
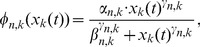
(3)where 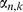, 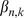 and 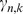 are tuning parameters.

In inferring the regulatory structure of GRNs consisting of several tens of genes, hill-function based differential equations, *e.g.*, Eqs. (1) and (2), become intractable. Therefore, we consider discretization and linearization of gene regulatory systems [8], [19]–[22], [27], [29], [44]. Here, linear functions are substituted for hill-functions and higher than quadratic terms are neglected. Furthermore, we assume that biological processes should include the effects by noise [47]. Let 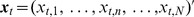 be a series of 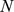 dimensional vectors containing expression levels of 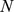 genes at the 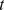
th time point. Then, we consider a gene regulatory system represented by
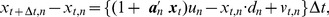
(4)where 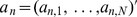 is an *N*-dimensional vector including regulatory effects on the *n*th gene by other genes, 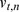 is the effects by noise at the *t*th time point, and 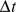 indicates a minute displacement. Then, a VAR model for GRNs simulation can be constructed.

In constructing gene regulatory models, we make an assumption that observational data are measured with observational noise. Under this assumption, to separately handle a system model (*i.e.*, Eq. (4)) and biological observational data, we utilize a state space representation [13], [21], [24], [29], [48]. Here, a minimum observational time step and 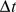 are usually handled as 1 for reducing computational cost, however, we can set any value for 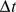 less than a minimum observational time step. Therefore, we evaluated the influence of changing 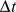 in the results section and describe the case of 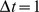 in the following for simplicity. Consequently, we consider a model described by
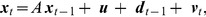
(5)

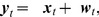
(6)

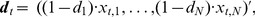
(7)where 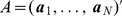 is an 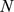 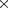 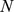 matrix representing regulation among genes, 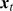 is an *N*-dimensional hidden state variable, 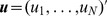 is an *N*-dimensional vector including synthesis rates, 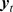 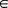 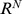 is a series of vectors containing observed expression levels of *N* genes at the *t*th time point and 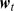 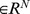 is observational noise. Here, we define a set of all points of time 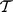 (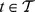), consisting of the observed time set 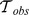 (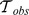 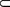 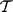). We set system noise 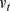 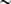 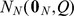 and observation noise 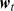 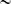 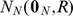, where 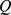 and 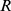 are *N*×*N* diagonal matrices. The initial state vector 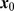 is assumed to be a Gaussian random vector with mean vector 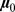 and covariance matrix 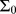, *i.e.*, 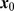 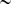 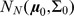. Note that 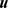 and 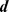 must be dense vectors; nevertheless, 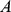 should be a sparse matrix, and activation and repression correspond to positive and negative values of 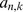, respectively, because hill-functions are monotonic.

**Figure 1:**
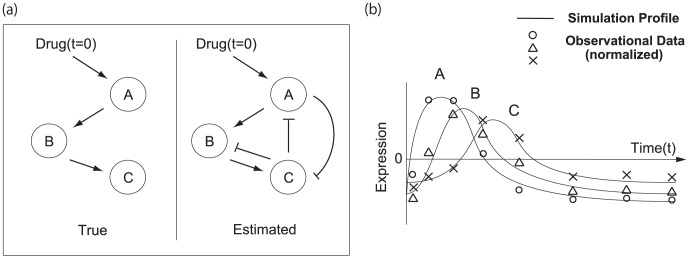
The problem of deleting a term representing a synthesis rate. A toy model indicating the problem of deleting a synthesis rate 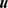 by shifting an average of observed time-course data for each element to 0, *i.e.*, 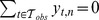 for 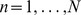 as a normalization procedure. The true network and the adjusted data are illustrated in the left panel in (a) and (b), respectively. As shown in the right panel in (a), some false positive edges are possibly estimated in comparison to the true relationships.

Contrary to the derivation of Eq. (5), in previous linear state space models for GRN analysis [21], [29], a simulation model was constructed as
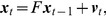
(8)where 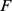 is an 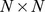 matrix in which the *n*th row and *k*th column element is represented by



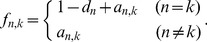
(9)In this model, 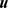 is removed by shifting the average of the observed time-course data for each element to 0, *i.e.*, 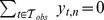 for 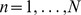, where 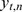 is the *n*th row element of 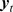, as a normalization procedure. However, this model may cause marked difficulty in estimating gene regulatory relationships if the observed time-course includes a steady state. Fig. 1 exemplifies such a situation.

Fig. 1 shows a small pathway consisting of three genes (left panel in Fig. 1 (a)) and the averages of the observed time-course data for each element are shifted to 0 (Fig. 1(b)). By applying Eq. (8) to the observed data, we expect to obtain three false edges added to the true pathway (right panel in Fig. 1(a)) because nodes must retain a constant steady state regardless of their negative steady state values and positive regulation from negative nodes. In some cases, such additional false regulation possibly hide true regulation. The above result encourages us to use a model explicitly implementing terms to represent a steady state of gene expressions to estimate gene regulatory relationships precisely. Furthermore, in using Eq. (8), when elements of 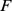 are regularized to be selected non-zero elements, even 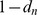 is regularized and 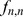 can be zero. To penalize the regulatory effect 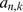 only, 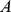 and 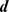 are separately described in our proposed model.

### Incorporation of Biomolecules Affecting Biological Systems

When simulating the dynamic behavior of GRNs including biomolecules that cannot be represented by 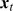 and can affect biological systems, *e.g.*, corticosteroids in corticosteroid-stimulated GRNs, we should consider the concentration of such biomolecules. For these cases, we remodel Eq. (5) to add a term representing the concentration of such biomolecules as
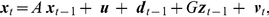
(10)where 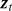 is an *M*-dimensional vector containing the concentration of the biomolecules at the *t*th time point, 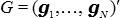 is an 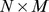 matrix and 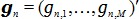 is an *M*-dimensional vector representing their regulatory effects on the *n*th gene. We consider the case that the concentration is known or can be simulated. In the results section, for an application example, we deal with corticosteroid drug pathways that have been well studied previously [37], [38], [41]; 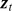 is given the concentration of the intra-nuclear corticosteroid-receptor complex employed in Yao *et al.* [38].

### State Space Model and Kalman Filter for Estimating the Hidden State

Recently, many types of state space models have been proposed and applied in the context of systems biology [13], [19]–[21], [23], [48], [49]. They are roughly divided into two major classes, *i.e.*, linear and nonlinear models. In using linear state space models, posterior probability densities of the hidden state can be obtained as Gaussian distributions and the optimal mean and covariance matrices can be analytically calculated by the Kalman filter algorithm [50], [51]. In contrast, for nonlinear state space models, because the analytical form can be intractable, several extensions of the Kalman filter algorithm, *e.g.*, extended Kalman filter [52], unscented Kalman filter [53], [54] and particle filter [55], which utilize approximation techniques, have been applied to obtain posterior probability densities of hidden state and parameters [13], [24], [48], [49], [56]. In using linear state space models [19]–[22], the main concern is to infer causal relationships among genes, for which regulatory structure is assumed to be sparse, *i.e.*, genes are regulated by only a few specific regulators. Imposing such a sparse constraint to regression approaches is a general problem, but for state space models to simultaneously estimate optimal hidden state and parameter values (including penalization parameters), it is not a trivial problem [27]–[30], [57]. Then, for example, a sparse regulatory structure was extracted by statistical tests after estimating parameter values [21]. In this article, under the framework of a state space representation of a VAR model, we intend to infer the parameter values and the hidden state maximizing prediction ability for observational data with a sparse regulatory structure. For this purpose, we apply the EM algorithm [58] in the next subsection and the conditional expectations of hidden state are given by using the Kalman filter algorithm.

### Kalman Filter Algorithm for VAR-SSM

Let 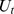 be the sum of 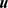 and 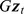. For simplicity, we here use *F* in Eq. (8) rather than *A*. The prediction, filtering, and smoothing of the Kalman filter are calculated by the following formulas:

- Prediction:

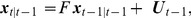
(11)

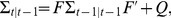
(12)

- Filtering:

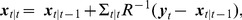
(13)

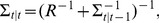
(14)

- Smoothing

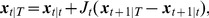
(15)

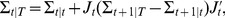
(16)

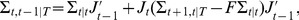
(17)

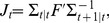
(18)

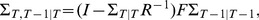
(19)where 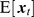 given 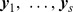 is represented by 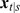 and 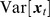 given 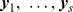 is represented by 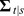. To calculate an inverse of the 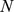 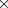 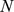 matrix, we use a matrix inversion theorem [29].

### Maximum Likelihood Estimation Using the EM Algorithm with L1 Regularization

In biological systems, most genes are regulated by a few specific genes, *i.e.*, 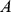 and 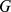 can be sparse matrices. Thus, we applied *L*1 regularization to select effective sets of elements for 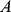 and 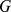. Let 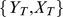 be the complete data set, where 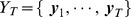 is the set of observed data and 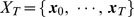 is the set of state variables. Furthermore, let the probability densities 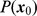, 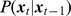 and 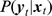 be the *N*-dimensional Gaussian distributions 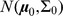, 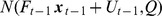 and 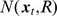, respectively. Then joint likelihood for the complete data set is given by
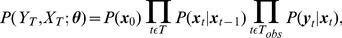
(20)where 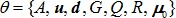. In this study, we used the EM algorithm [58] to search for the parameter vector 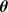 that maximizes 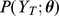 under *L*1 regularization. The *L*1 regularized log-likelihood is given by
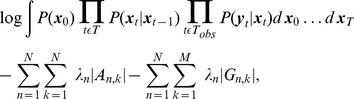
(21)where 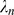 is the *L*1 regularization term for the *n*th row. In the EM algorithm, the conditional expectation of the joint log-likelihood of the complete data set
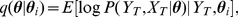
(22)is iteratively maximized with respect to 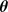 until convergence, where 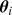 is the parameter vector obtained at the *i*th (previous) iteration.

The detailed solution for estimating parameter values using the EM algorithm for VAR-SSM with *L*1 regularization can be found in Method S1.

### Parameter Optimization Algorithm with *L*1 Regularization

Because of the combination of the regularization terms and a state space representation, updating an element of 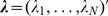 influences the other active sets. Thus, it is difficult to select the optimal active sets 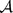 and 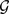, the values of 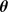 and 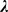 at the same time. Therefore, we proposed a novel algorithm to separately update 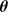 and 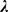 in each row as follows. In this algorithm, we consider candidates of active sets for 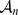 and 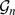 as 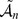 and 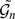, respectively. In the EM algorithm in Method S1, we constraint that the active sets 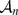 and 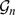 can be selected from 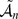 and 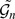, respectively, *i.e.*, 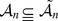 and 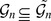


### Algorithm

-Initial Settings
  1. Set 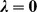 and recursively update 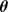 to obtain 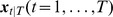 using the EM algorithm until convergence is attained. In this step, active sets 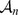 and 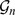 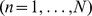 consist of all elements, *i.e.*, 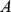 and 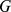 become dense matrices, since the regularization terms can be neglected. Thus, the solution of the EM algorithm is directly obtained from Eqs. (SI–11)–(SI–17) in Method S1.
  2. Set the maximum number of iterations to be 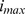, the maximum number of regulatory edges for each gene to be 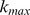 and 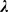 to be sufficiently high to allow all elements of 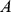 and 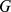 to become 0, and 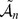 and 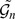 to be full. Alternatively, 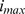 can be set as a value when the Bayesian information criterion (BIC) [59]–[61], which are used to select the best model in this algorithms, is not updated through iterations and 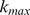 can be set a sufficiently high value, *e.g.*, 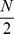. The BIC score in this algorithm is defined as

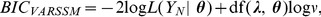
(23)

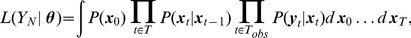
(24)where 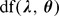 is the degree of freedom, *i.e.*, the number of active parameters [61], and 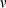 is the number of samples.

-
  3. Set 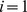 and recursively update 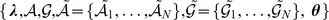 as follows. Note that, at 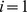, we fix 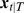 as the values obtained at Step 1, except for the updating elements indicated as 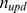 in the next step. Thus, we only update the values of the parameters for the 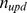
th row at 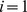.
-Main Routine
  4. For 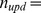 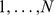

    - (a) Set 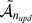 and 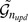 full and 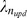 sufficiently high to allow all elements of 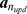 and 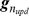 become **0**. Through the following steps, fixing 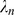 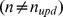, 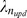 is gradually decreased to find an optimum 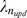 for which the BIC score is minimized.
    - (b) Calculate conditional expectations using the Kalman filter.
      (c) Update 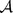, 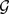, and 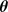 by Eqs. (SI–11)–(SI–19) in Method S1. Here, 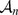 and 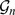 of Eqs. (SI–18)–(SI–19) in Method S1 can be constructed from 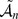 and 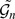, respectively.
      (d) Calculate the BIC score and decrease 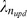 if the regularized log-likelihood of Eq. (21) is converged. Then, repeat from step (b) until the sum total of 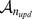 and 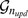 becomes 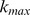.
      (e) Set 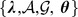 as the value with the lowest BIC score obtained through the above described steps. Furthermore, set 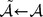 and 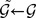.
      (f) Consider the set of all subsets of 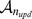 and 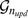 as 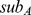 and 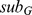, respectively. For all 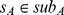 and 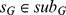, setting 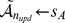 and 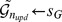, repeat steps 4(b) and (c), and then obtain the BIC scores of converged log-likelihood.
      (g) Set 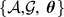 as the value with the lowest BIC score. Furthermore, 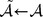 and 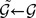.
  5. Set 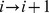 and repeat from step 4 until *i* becomes 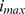.

**Figure 2:**
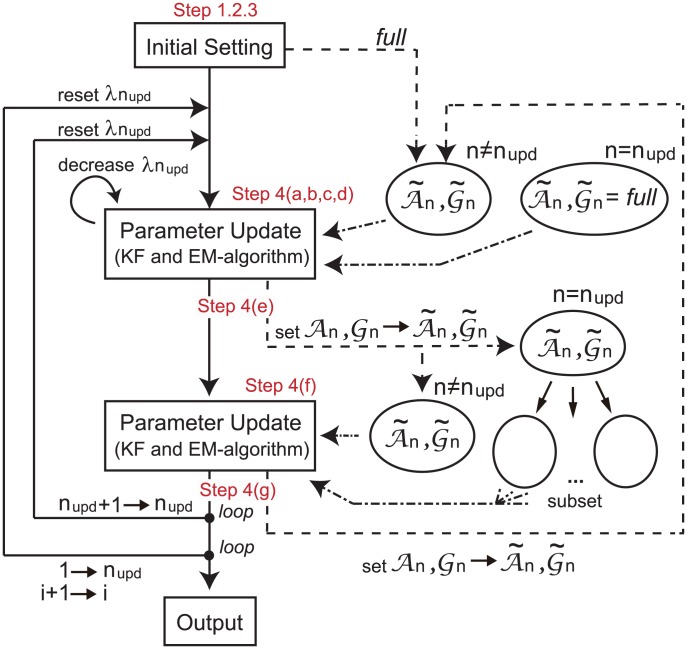
The conceptual view of the proposed algorithm. This figure illustrates a conceptual view of the proposed algorithm. The notations ‘Step’ correspond to those of the proposed algorithm. Solid, dashed and chain lines represent flowchart of the algorithm, setting the parameter values and active sets, and setting candidates of active sets used for selecting active sets.

A conceptual view and a pseudo code of the algorithm are shown in Figure 2 and Algorithm 1 in Table 1, respectively. We should note that, since the active sets 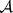 and 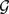 obtained at step 4(e) may not be the optimal ones for the selected 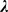, *i.e.*, there can exist better ones having lower BIC scores for the selected 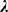, the proposed algorithm further explores such better ones by evaluating subsets of the obtained active sets at step 4(e) through steps 4(f) and (g).

**Table 1:** Algorithm 1: A pseudo code of Main Routine (step 4 and 5) in the proposed algorithm.

1: 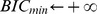 ;
2: **for** 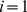 to 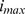 **do**
3: **for** 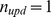 to 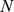 **do**
4: 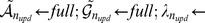 a sufficiently high value;
5: **while** 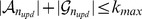 **do**
6: **while** convergence criterion is not satisfied **do**
7: Update 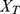 and parameter values using the Kalman filter and the EM-algorithm;
8: **end while**
9: **if** 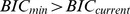 ; **then**
10: 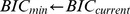 ; Store the current parameter values;
11: where 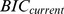 is the BIC score of the current parameter values
12: **end if**
13: Decrease 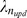 ;
14: **end while**
15: Set the stored parameter values as the current parameter values;
16: 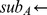 the set of all subsets of the current 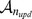 ;
17: 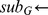 the set of all subsets of the current 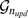 ;
18: **for all** 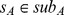 **do**
19: 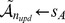 ;
20: **for all** 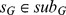 **do**
21: 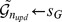 ;
22: **while** convergence criterion is not satisfied **do**
23: Update 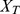 and parameter values using the Kalman filter and the EM-algorithm;
24: **end while**
25: **if** 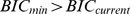 **then**
26: 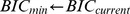 ; Store the current parameter values;
27: **end if**
28: **end for**
29: **end for**
30: Set the stored parameter values as the current parameter values;
31: **end for**
32: **end for**

### Weighting Known Regulations

To weight parameters of known regulations, *e.g.*, as recorded in the literature, we derive the weighted regularization [62]. For the *n*th row, we define the weight vectors 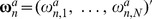 and 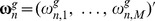. The elements of these vectors for known regulations are set to less than 1 or, otherwise set to 1. Then, in the M step of the EM algorithm and the regularized log-likelihood, regularization terms are handled as
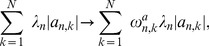
(25)




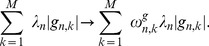
(26)


In practice, the purpose of the weight is to select known regulation in the instance where multiple candidates are highly correlated with the same gene. Thus, when the correlation of a known regulation is still a low value, the regulation should not be selected as an active regulation. For example, weights for literature-recorded pathways and regulations by TFs are set as 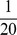 and 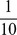 in the real data experiment, respectively. The effectiveness of the weighted regularization is demonstrated in the results section.

## Results

### Comparison Results

To show the effectiveness of the proposed method, we compared it with other GRN inference methods, *i.e.*, a state space model (SSM) [21], [63], a general VAR model using the LARS-LASSO algorithm [30], [61], GeneNet [31], [32] based on an empirical graphical Gaussian model (GGM), dynamic Bayesian networks using first order conditional dependencies (G1DBN) [33], GLASSO [34] based on sparse GGM and the mutual information-based network inference algorithms: ARACNE [3], CLR [35] and MRNET [36]. We applied these inference methods by using R-package (‘GeneNet’, ‘G1DBN’, ‘glasso’ and ‘parmigene’) and implementing the others. The comparison analysis was performed using three artificial data, which were generated based on pharmacogenomic pathways that we assumed and a yeast network that was produced as a part of the DREAM4 (Dialogue for Reverse Engineering Assessments and Methods) challenge. We should note that, because ARACNE, CLR and MRNET are intended to infer static relationships between genes, we considered time-course observational data as static data utilizing a time-lag matrix, in which the *t*th row vector consists of 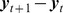, according to Shimamura *et al.* [57]. Note that the Jar file of the proposed method is available at: http://sunflower.kuicr.kyoto-u.ac.jp.

### Comparison Using Pharmacogenomic Pathways

For the comparison, we first generated two time-courses from (i) linear difference equations as Eq. (4) and (ii) nonlinear differential equations as Eqs. (1)–(3) representing pharmacogenomic pathways (*e.g.*, Yao *et al.* [38]) using Cell Illustrator 5.0 (http://www.cellillustrator.com/home). The details of the artificial simulation models are as follows.

**Figure 3:**
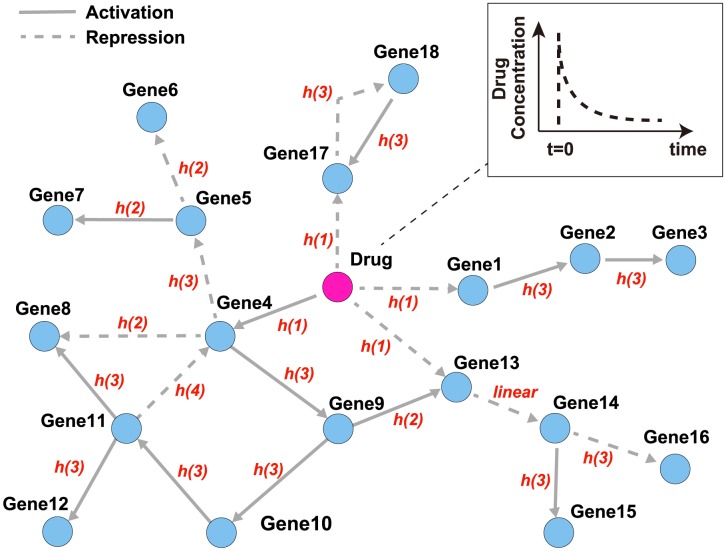
A pharmacogenomic pathway of the artificial simulation model. The figure illustrates the pathway of the artificial simulation model used for datasets (i) and (ii). Each regulation is represented by (i) a linear and (ii) a nonlinear function, such as Eq. (3). For dataset (ii), descriptions on edges as 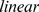 or 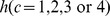 means a linear function and a hill function, described in Eq. (3) when 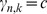, respectively. The system is stable at first (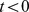) and undergoes stimulation by a drug at 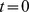. The concentration of the drug is gradually decreased according to the drug kinetics. A solid arrow and a dotted arrow mean activation and repression, respectively.

**Figure 4:**
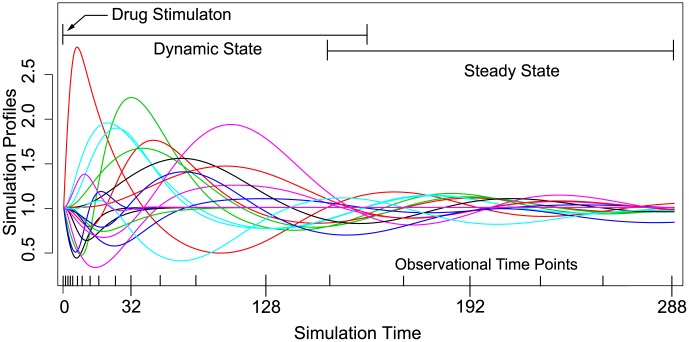
The simulation expression profiles of genes of the artificial simulation model. This illustrates the simulation expression profiles of genes of the artificial simulation model used for dataset (ii). The simulated data for datasets (i) and (ii) have both dynamic and steady state, and stimulated by the drug at 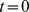. Observational time-course data is obtained with Gaussian noise from the simulation expression at the time points that are indicated on the bottom axis. The observational data, parameter values and simulation models are available at Models S1 and S2.

*-Dataset*(*i*)
  1. The number of genes is 18.
  2. Each gene undergoes synthesis and degradation processes, and genes are mutually regulated as shown in Fig 3 (The details of the figure are explained below).
  3. A drug is added at 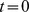 and its concentration gradually decreases according to one compartment model, *i.e.*, 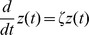, where 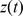 is the concentration of the drug as a function of time 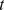 and 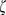 is the degradation rate. The simulated expression profiles of the genes are initiated by the drug at 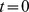 and gradually converge to their steady states as illustrated in Fig. 4.
  4. The expression data is observed at 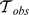 = 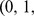 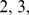 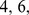 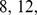 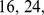 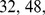 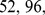 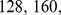 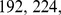 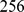 and 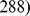 with Gaussian observation noise of mean 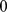 and a variance that is proportional to the intensity.
  5. The number of replicated observations with different observational noise for each time point is three.
  6. The simulated expression is updated according to the linear difference equations represented by Eq. (4) at 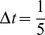.
  7. The observational data and the values of the parameters are available at Model S1.
*-Dataset*(*ii*)

1 to 5 of dataset (i) are also satisfied in dataset (ii).

  6. The simulated expression is updated according to the differential equations. Regulatory relationships are the same as in (i) but the regulatory effects are represented by hill functions, such as Eqs. (1)–(3), or linear functions, as illustrated in Fig 3. In this figure, 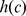 indicates that the regulation is described by Eq. (3) when 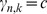.
  7. The observational data and the csml (cell system markup language) file are available at Model S2.

**Figure 5:**
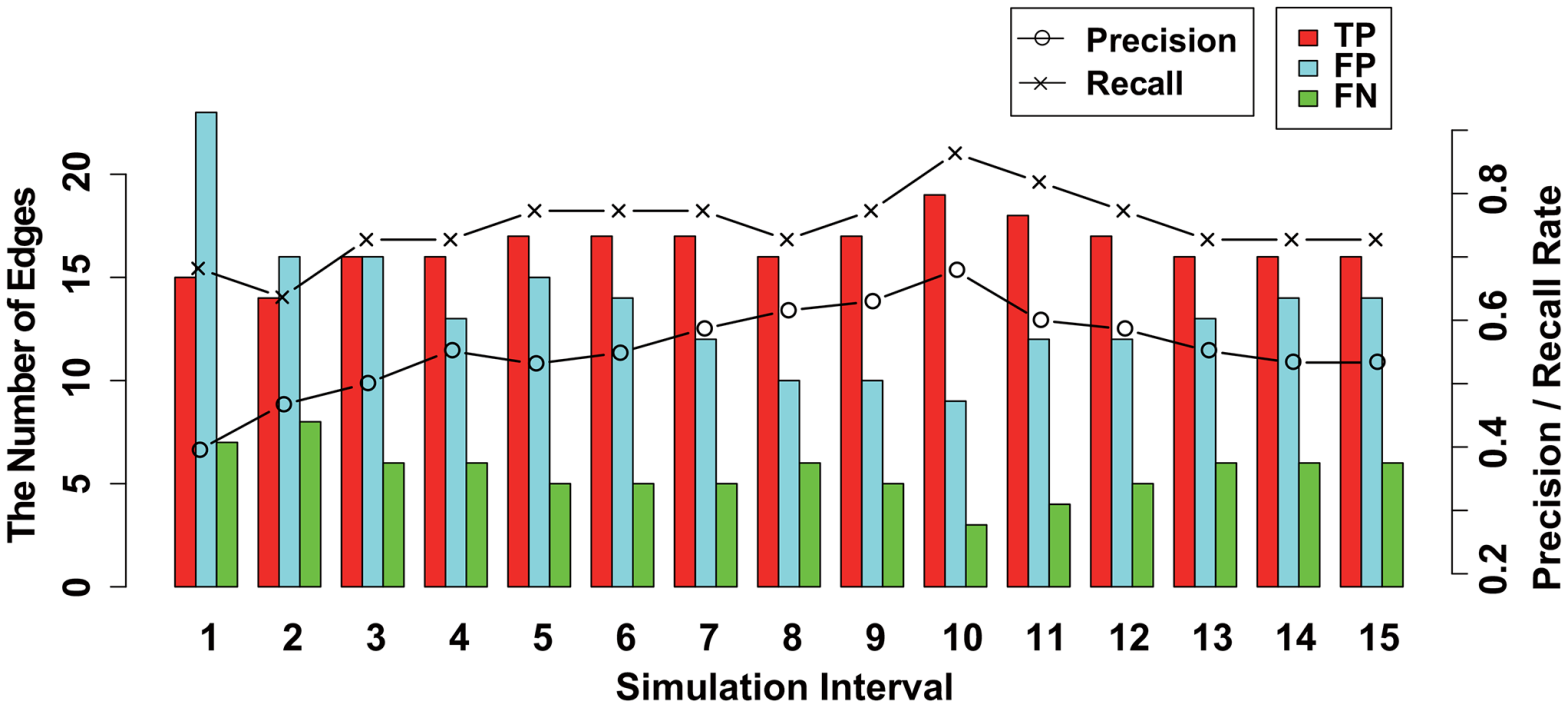
The results of the structure inference using dataset (i) of a pharmacogenomic pathway by the proposed method. This figure illustrates the results of the structure inference after applying the proposed method to dataset (i) for each simulation time interval 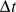. The histogram represents the number of true positive (TP), false positive (FP), and false negative (FN) findings for each 
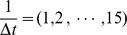
 as red, blue, and green bars, respectively. Black lines with circles and crosses represent ‘precision rate (PR = 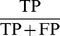)’ and ‘recall rate (RR = 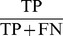)’, respectively. The values of the histogram and lines correspond to the left and right axes, respectively.

**Figure 6:**
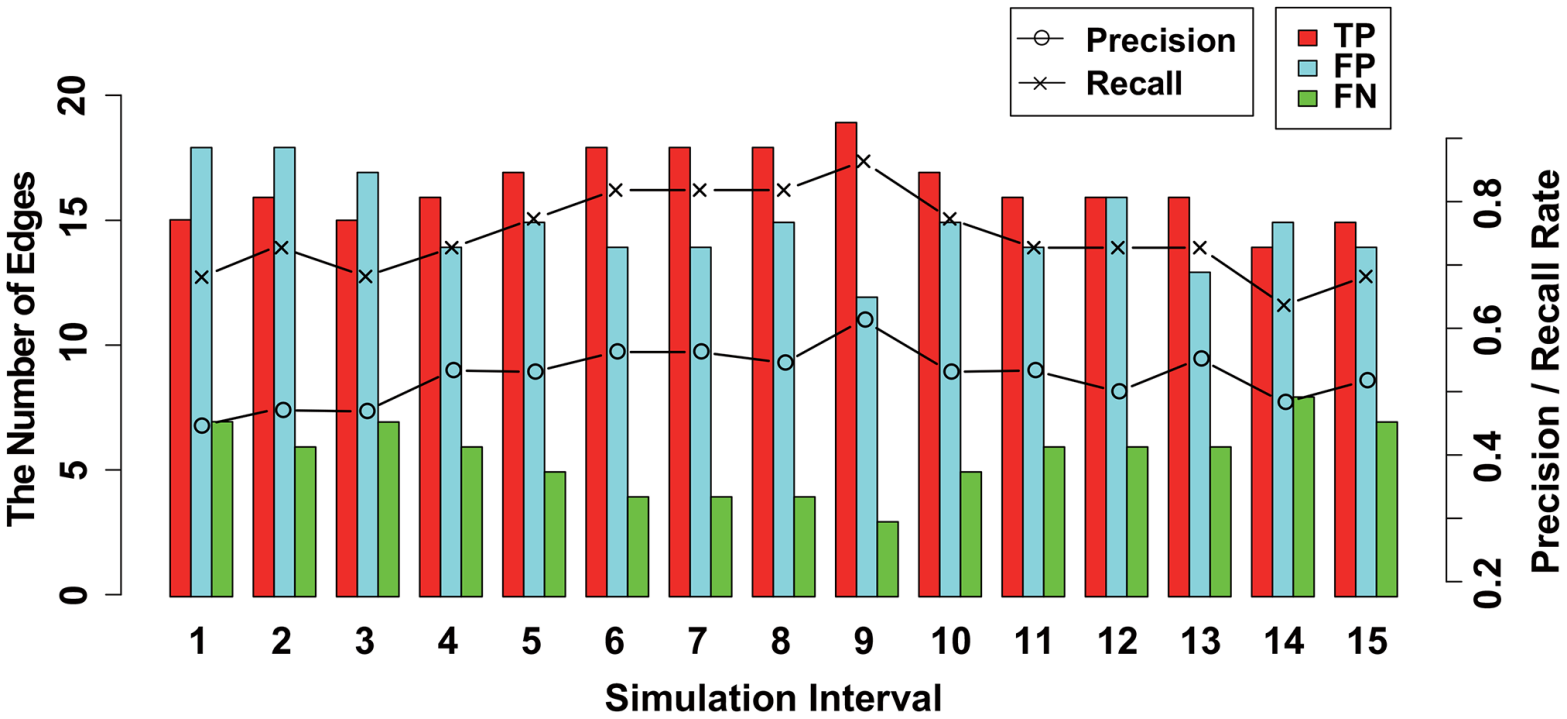
The results of the structure inference using dataset (ii) of a pharmacogenomic pathway by the proposed method. This figure illustrates the results of the structure inference after applying the proposed method to dataset (ii) for each simulation time interval 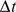. The histogram represents the number of true positive (TP), false positive (FP), and false negative (FN) findings for each 
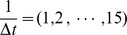
 as red, blue, and green bars, respectively. Black lines with circles and crosses represent ‘precision rate (PR = 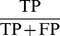)’ and ‘recall rate (RR = 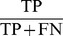)’, respectively. The values of the histogram and lines correspond to the left and right axes, respectively.

**Figure 7:**
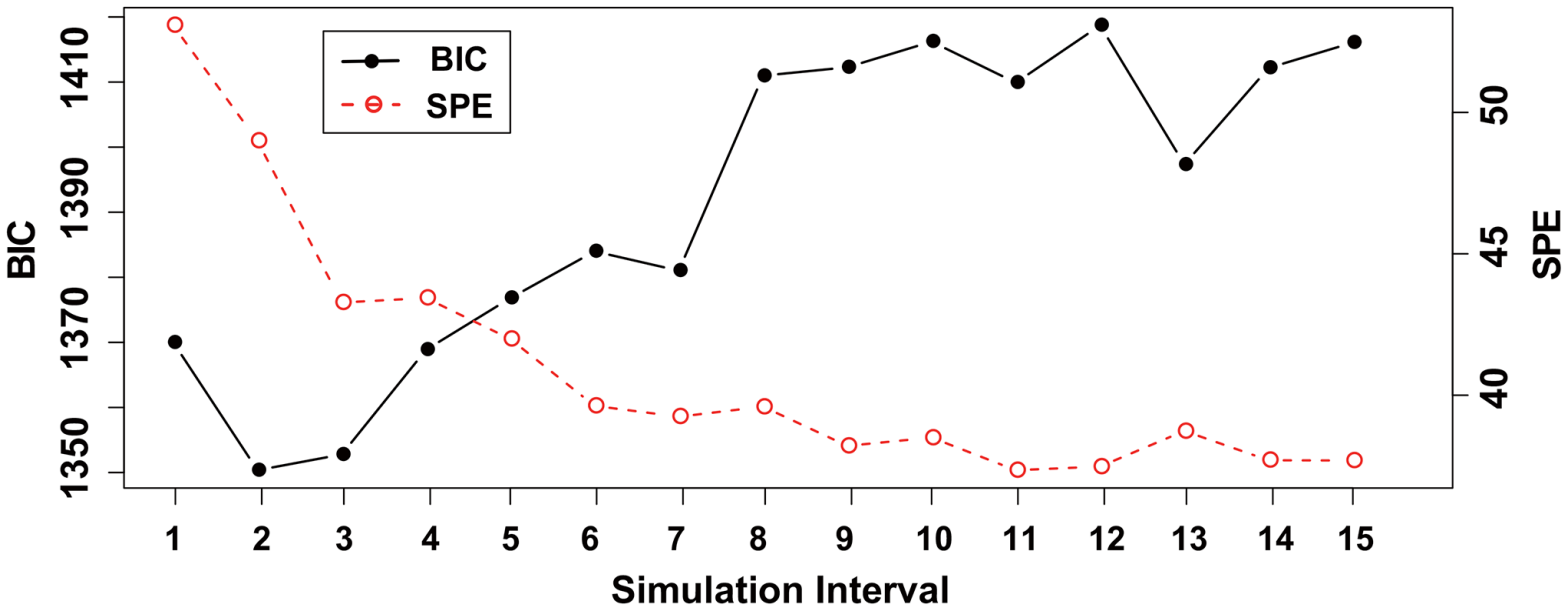
The result of the BIC scores and SPE for each simulation time interval using dataset (i). This illustrates the BIC scores and SPE (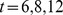) for 
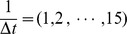
 for dataset (i). The values of the BIC scores and SPE correspond to the left and right axes, respectively.

**Figure 8:**
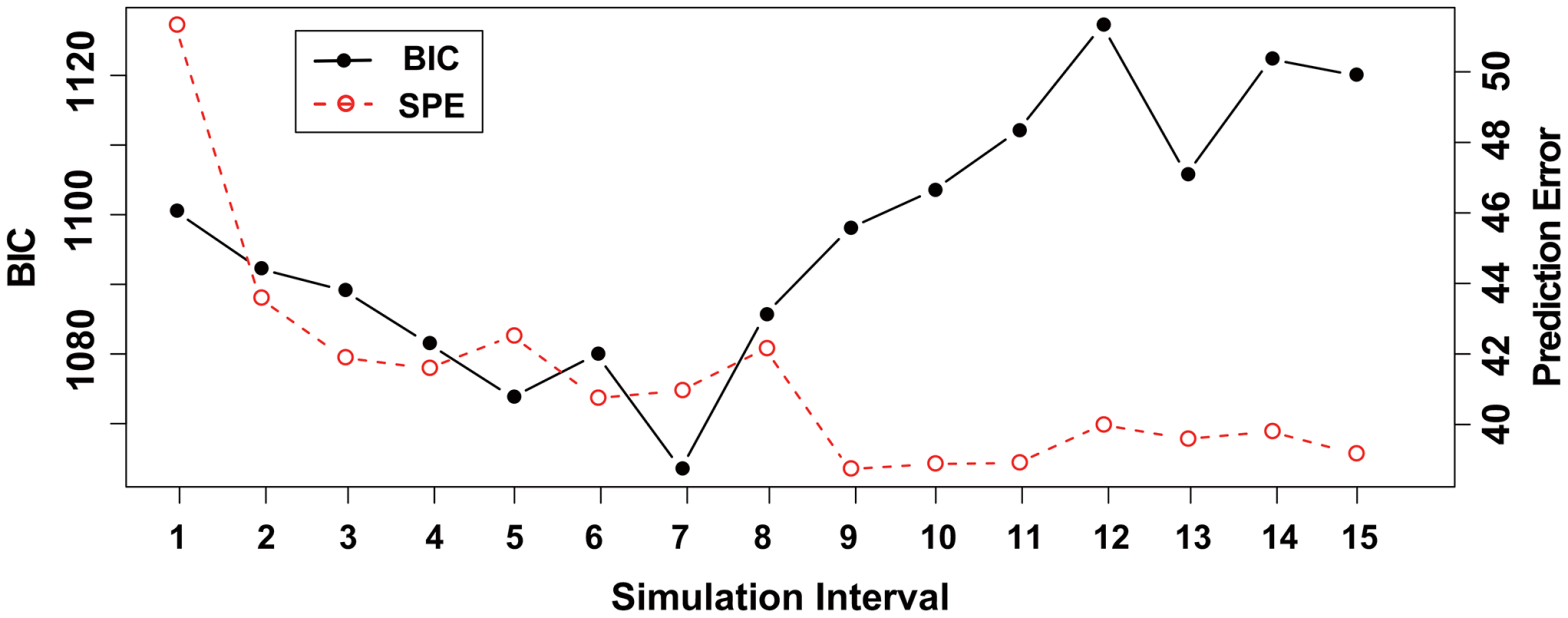
The result of the BIC scores and SPE for each simulation time interval using dataset (ii). This illustrates the BIC scores and SPE (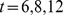) for 
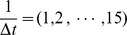
 for dataset (ii). The values of the BIC scores and SPE correspond to the left and right axes, respectively.

A true positive (TP), false positive (FP), false negative (FN), precision rate (PR = 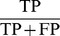), and recall rate (RR = 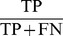) were used to measure the performance. At first, in applying the proposed method to the data, we changed the simulation time interval of Eq. (4) to 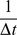 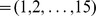, and estimated active sets of regulation (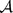 and 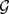) and the values of the parameters for each 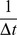 for each dataset. The results for datasets (i) and (ii) are illustrated in Figs. 5 and 6, respectively. The precision and recall rates in Figs. 5 and 6 show that the performance of the structure inference gradually increases from 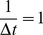 and is optimal at 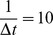 for (i) and 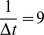 for (ii). This indicates that the simulation time interval 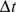 can influence the performance of structure inference and we should carefully design 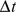 for biological simulations. In order to determine 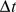, we measured the BIC scores and the sum of squared prediction errors (SPE) at three time points (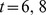 and 12) for each 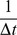 using (i) and (ii), as represented in Fig. 7 and Fig. 8, respectively. Here, we measured the prediction errors for each time point by optimizing the values of the estimated parameters without using the observational data at the corresponding time point (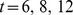).

For dataset (i), although the PR and RR values peak at 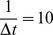, the BIC scores become lowest at 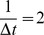. Similarly, the BIC score becomes lowest at 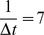 but peaks at 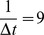 for dataset (ii). SPE gradually converges when 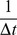 becomes large and has the lowest value at 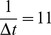 and 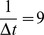 for datasets (i) and (ii), respectively. Therefore, SPE can be an indicator for determining the best time interval for this hill function-based system of pharmacogenomics. Note that the measured time points for the prediction errors should be the points that are not steady state values.

Next, we compared the results of (a) the proposed VAR-SSM with the lowest BIC and (b) the proposed VAR-SSM with the lowest SPE to (c) SSM [21], [63] (permutation tests were utilized to select regulations), (d) VAR model with *L*1 regularization using the LARS-LASSO algorithm [30], [61] (the BIC score is used to determine the value of the regularization parameters), GeneNet [31], [32], G1DBN [33], GLASSO [34], ARACNE [3], CLR [35] and MRNET [36]. The comparison results for datasets (i) and (ii) are listed in Tables 2 and 3, respectively. In these comparisons, we added the drug profiles to the observational data and did not count regulations in response to drugs and self-regulation. For the methods inferring undirected regulations, *i.e.*, GeneNet, GLASSO, ARACNE, CLR and MRNET, we considered the true network (directed network) as an undirected network and then measured the performance by comparing this undirected network to the inferred networks. Additionally, for GeneNet, G1DBN and mutual information-based methods (ARACNE, CLR and MRNET), which are required to set a threshold value to determine the existence of regulation, we checked the results of setting the threshold *q*-value (GeneNet) and posterior probability (G1DBN) to 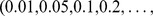 and 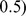 and a cut-off value (ARACNE, CLR and MRNET) to 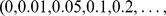 and 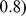, and adopted the best thresholds with respect to *F*-
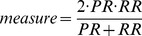
. We should note that the simulation time interval of SSM is set 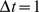 (no other choice is available) due to the implementation of Tamada *et al.* [63]. It is hard to make the simulation time interval short; hence, the simulated expression profiles often oscillated in such situations.

**Table 2:** Comparison of the proposed method and the existing methods using dataset (i).

	PR	RR	TP	FP	TN	FN
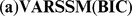	0.467	0.634	14	16	286	8
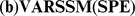	0.600	0.818	18	12	290	4
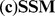	0.308	0.182	4	9	293	18
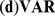	0.150	0.773	17	97	205	5
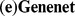	0.280	0.667	14	36	114	7
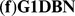	0.314	0.500	11	24	278	11
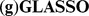	0.094	0.286	6	58	92	15
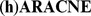	0.131	0.524	11	71	79	10
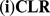	0.135	0.619	13	83	67	8
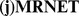	0.121	0.571	12	87	63	9

**Table 3:** Comparison of the proposed method and the existing methods using dataset (ii).

	PR	RR	TP	FP	TN	FN
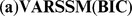	0.563	0.818	18	14	288	4
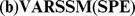	0.613	0.864	19	12	290	3
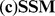	0.234	0.318	7	23	279	15
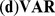	0.206	1.000	22	84	236	0
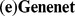	0.278	0.714	15	39	111	6
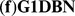	0.647	0.500	11	6	296	11
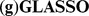	0.052	0.143	3	55	95	18
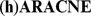	0.191	0.429	9	38	112	12
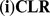	0.156	0.667	14	76	74	7
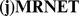	0.156	0.667	14	76	74	7

**Figure 9:**
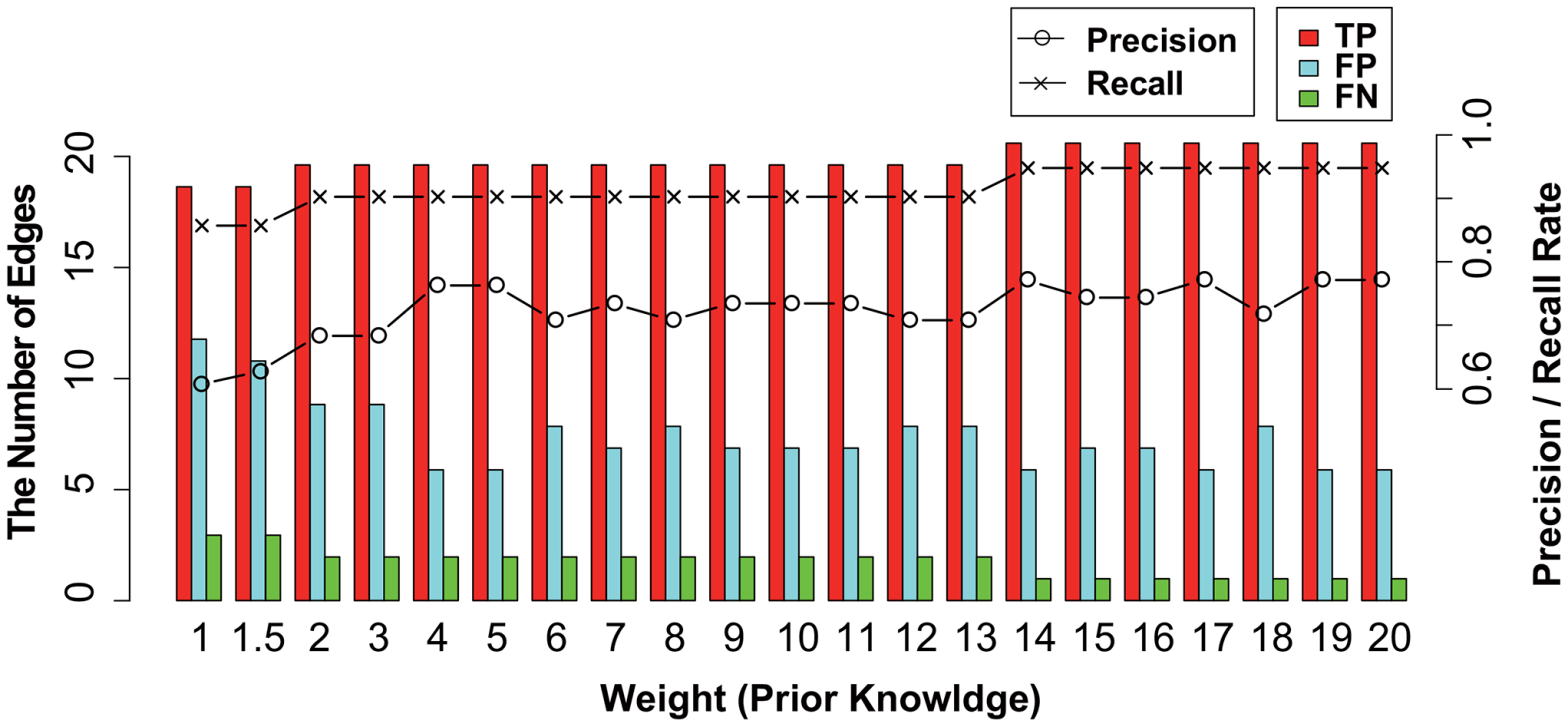
The performance of using prior knowledge as the weighted regularization. This figure illustrates the effectiveness of the weighted regularization (prior knowledge) at simulation time interval 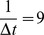 using dataset (ii). The histogram represents the number of true positive (TP), false positive (FP), and false negative (FN) findings for each 
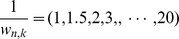
 as red, blue, and green bars, respectively. Black lines with circles and crosses represent ‘precision rate (PR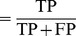)’ and ‘recall rate (RR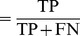)’, respectively. The values of the histogram and lines correspond to the left and right axes, respectively.

Consequently, the proposed method achieved a low false positive rate while maintaining a high true positive rate. These results may be acceptable because the system model of the proposed method is the same as or similar to the artificial simulation models. Thus, it is conceivable that the proposed method is highly capable of inferring the regulatory structure of the assumed hill-function based model. Furthermore, we demonstrated the effectiveness of the weighted regularization for known prior information using dataset (ii). To evaluate the performance, we adapted a simulation time interval of 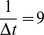. Setting weights for true regulations as 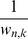 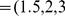, 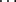, 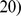, PR and RR were evaluated as illustrated in Fig. 9. The correct weights reduced the FP and FN edges, and the performance was gradually improved according to the increase in the weight coefficient. In contrast, several FP edges still exist even when the weight coefficients take on high values. It can be considered that the simplification of the true regulatory system using the proposed model generates these false edges to effectively predict the data.

### Comparison Using Yeast Network of a Part of the DREAM4 Challenge

In contrast to the previous comparisons, for which the data were based on the assumed models as Eqs. (1)–(4), we next prepared data generated by GeneNetWaver [39], [40] using a 10-node yeast network (*yeast 1*) of a part of the DREAM4 challenge (in silico network challenge). To measure the performance of the proposed method, in this comparison, we generated dataset (iii), which was a set of 100 time-course observational data, in which the measured time points were 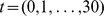.

According to the original setting, three genes, which were randomly selected for each time-course, were perturbed among 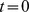 to 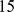. Here, since we intended to consider the case that observational data have a steady state, the number of time points was to be set larger than those of the original setting 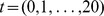. The dataset (iii) is available at Model S3.

**Figure 10:**
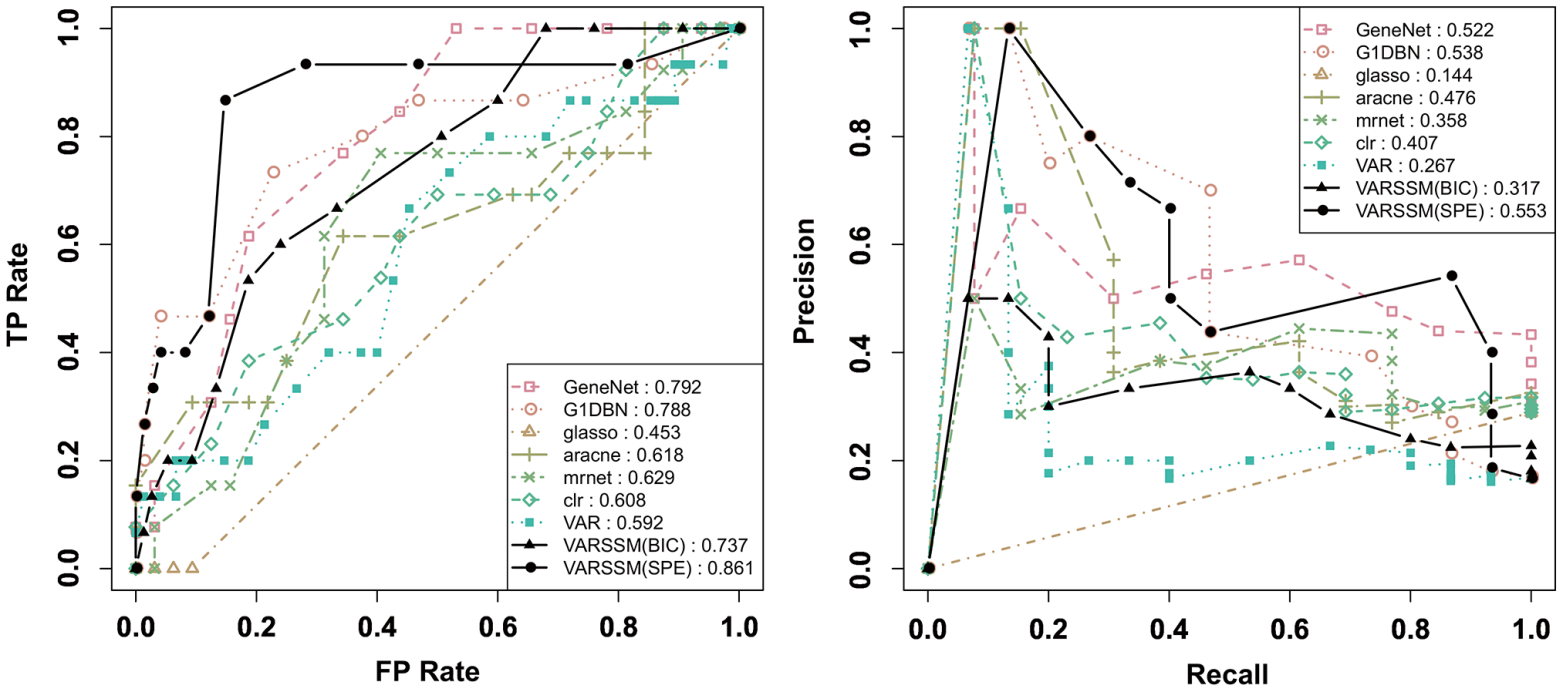
The ROC and PR curves using dataset (iii). The left and the right figures illustrate the ROC and PR curves for dataset (iii), respectively. In the left figure, the vertical axis and horizontal axis correspond to TP rate and FP rate, respectively. In the right figure, the vertical axis and horizontal axis correspond to PR and RR, respectively. AUROC and AUPR are represented at the right side of the inference methods.

We applied the methods (a)–(j) to dataset (iii); however, since SSM [21], [63] requires large computational costs to perform permutation tests for each time-course, we neglected SSM for this comparison. The time points to calculate SPE for the proposed method are 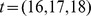, which are the time points shortly after removal of perturbations. For each method, we summed the existence of the estimated regulation on the *i*th gene by *j*th gene as 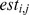 and considered the values 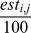 as the confidence level for the regulation. Then, TP rate (TPR = 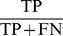), FP rate (FPR = 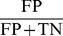), precision rate (PR = 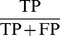) and recall rate (RR = 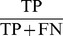) were calculated to draw ROC and PR curves. Using these curves, we measured the performance with respect to the AUROC (area under the ROC curve) and AUPR (area under the PR curve). These comparison results are illustrated in Fig. 10. Note that, similarly to the previous experiments, we selected the best threshold values with respect to AUROC for the methods (e), (f) and (h)–(j).

As a result, although the simulation model for dataset (iii) is different from the models that we assumed, the proposed method using SPE outperformed the other methods in terms of both AUROC and AUPR. The number of selected simulation time intervals 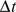 is shown in Table 4. These results indicate that the proposed method has good ability for inferring the regulatory relationships using time-course observational data for which regulations are not based on the model that we assumed. Furthermore, we can consider the SPE as a good indicator for determining the simulation time interval.

**Table 4:** The number of selected simulation time intervals for dataset (iii).

	1	2	3	4	5	6	7	8	9	10	11	12	13	14	15
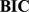	99	0	0	0	0	1	0	0	0	0	0	0	0	0	0
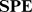	3	13	9	1	4	0	2	6	5	6	3	11	6	12	19

### Application to Corticosteroid Pathways in Rats

As an application example, we analyzed microarray time-course gene expression data from rat skeletal muscle [37], [38], which is assumed to have the same system used in simulation studies. The microarray data were downloaded from the GEO database (GSE490). The time-course gene expression was measured at 0, 0.25, 0.5, 0.75, 1, 2, 4, 5, 5.5, 7, 8, 12, 18, 30, 48, and 72 [h] (16 time points) after the glucocorticoid was applied. The data at time 0 represent controls (untreated). There were two, three, or four replicated observations for each time point.

Because corticosteroid pharmacokinetics/dynamics in skeletal muscle have been modeled based on differential equations [38] as shown in Model S4, the time-dependent concentration of corticosteroid in rat skeletal muscle can be obtained as 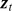. Furthermore, corticosteroid catabolic/anabolic processes in rat skeletal muscle have been partly established [41]; thus, these regulatory relationships can also be used. Given this information, we included *Mtor*, *Anxa3*, *Bnip3*, *Bcat2*, *Foxo1*, *Trim63*, *Akt1*, *Akt2*, *Akt3*, *Rheb*, *Igf1*, *Igf1r*, *Pik3c3*, *Pik3cd*, *Pik3cb*, *Pik3c2g*, *Slc2a4*, and *Mstn*. Note that the microarray (GSE490) does not include three genes in the original pathway [41], *Redd1*, *Bcaa* and *Klf15*. In addition, we employed the genes, *Irs1*, *Srebf1*, *Rxrg*, *Scarb1*, *Gpam*, *Scd*, *Gpd2*, *Mapk6*, *Ace*, *Ptpn1*, *Ptprf*, *Edn1*, *Agtr1a*, *Ppard*, *Hmgcs2*, *Serpine1*, *Cebpb*, *Cebpd*, *Il6r*, *Mapk14*, *Ucp3*, and *Pdk4*, which have been suggested to be corticosteroid-induced genes [37]. In summary, we applied the method to these 40 genes with weights for the established pathway and the concentration of corticosteroid.

**Figure 11:**
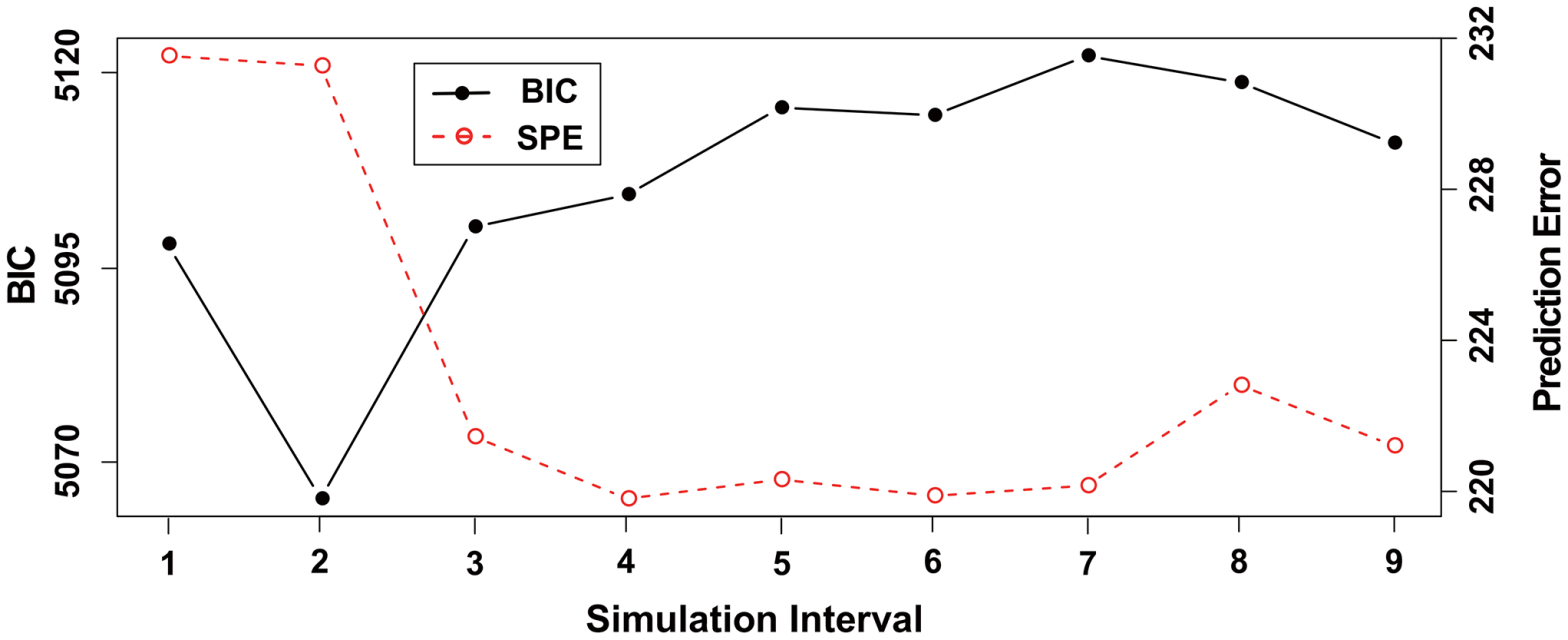
The result of the BIC scores and SPE for each simulation time interval using the real data. This illustrates the BIC scores and SPE 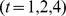 for each time interval 
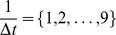
. The values of these indicators corresponds to the left and right axes, respectively.

**Figure 12:**
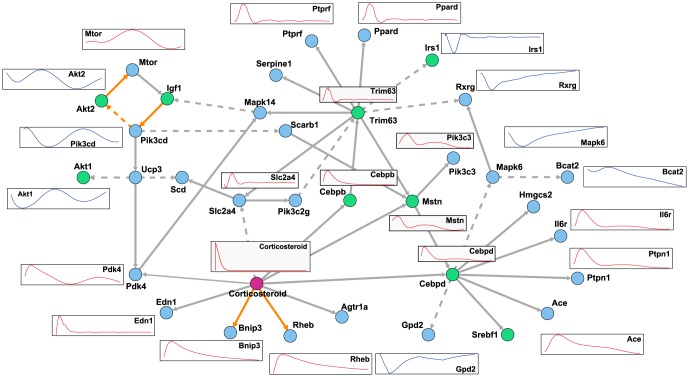
The estimated network with weighting literature-recorded pathways. This figure illustrates the inferred gene regulatory network with weights for literature-recorded pathways. *Corticosteroid* and genes of TFs are drawn as a red circle and green circles, respectively. Estimated edges with weights are illustrated as orange. Further, on some genes, simulation expression profiles are attached as examples. Red and blue profiles are roughly distinguished to up-regulated and down-regulated genes, respectively.

**Figure 13:**
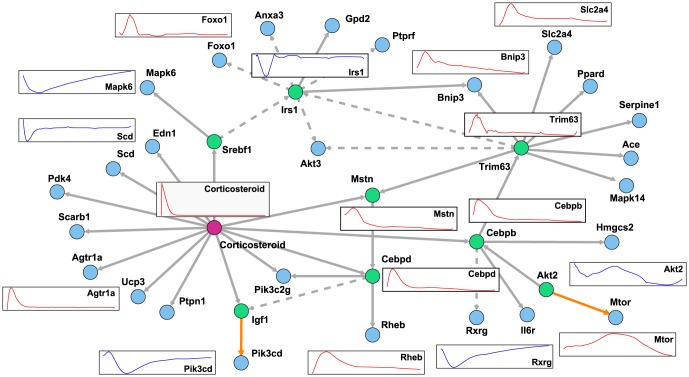
The estimated network with weighting literature-recorded pathways and regulations by TFs. This figure illustrates the inferred gene regulatory network with weights for literature-recorded pathways and regulations by TFs. *Corticosteroid* and genes of TFs are drawn as a red circle and green circles, respectively. Estimated edges with weights for literature derived regulations are illustrated as orange. Red and blue simulation profiles are roughly distinguished to up-regulated and down-regulated genes, respectively.

First, to determine the simulation time interval from 
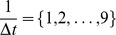
, we evaluated the BIC scores and SPE (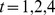). The results are shown in Fig. 11. Interestingly, even for the observational data, we obtained the same tendency for both indicators. Therefore, we obtained 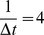 for the lowest SPE. Next, we analyzed the result of 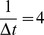. The inferred structure with some simulated expression profiles are illustrated in Fig. 12. From the figure, we can capture the propagation of gene expression stimulated by *corticosteroid* and hub genes regulating other genes. However, these results may be difficult to biologically interpret because some mRNAs are not considered to regulate other genes. Therefore, to exploit biological meaning correctly and demonstrate the effectiveness of incorporating prior information in the case of real biological data, we finally performed an experiment using TF information from ITFP (Integrated Transcription Factor Platform) [42]. Then, weights for regulations by TFs, *Trim63*, *Akt1*, *Akt2*, *Mstn*, *Irs1*, *Srebf1*, *Gpam*, *Cebpb*, and *Cebpd*, were set 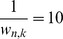. The inferred structure at 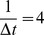 using the TF information is illustrated in Fig. 13.

In Figs. 12 and 13, there are some interesting observations. At first, some genes are directly regulated by corticosteroids, which are included in the model as 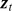. Thus, other models that do not include the drug terms cannot estimate such regulation. Second, only weighted regulations, *i.e.*, literature-recorded pathways and regulation by TFs, were inferred in contrast to the non-weighted network in Fig. 12. Thus, we could successfully incorporate prior knowledge, and further candidates may extend our understanding of regulation not yet reported in literature. Additionally, some weighted genes, *Cebpb*, *Mstn*, *Cebpd*, and *Trim63*, were also selected as hub genes with no weight in Fig. 12. Third, *Cebpb*, which is known as a transcription factor related to immune and inflammatory responses, is indicated as a hub gene (illustrated as a green circle). *Cebpd* and *Cebpb* are assumed to be candidate genes for insulin-related transcription factors [64]. This finding may confirm the findings of previous studies [37], [38] indicating that corticosteroid stimulation of skeletal muscle can induce the expression of insulin.

Finally, we applied the other methods, *i.e.*, GeneNet and G1DBN, to the pharmacogenomic data and attached significance levels (*q*-val and 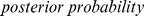 for GeneNet and G1DBN, respectively) for the regulations inferred by the proposed method. The results are presented in Table 5. Interestingly, some regulations have very high significance levels but others do not. For example, regulations of *Srebf1*, *Agtr1a*, *Cebpb* and *Cepbd* by a *corticosteroid* are quite probable. In contrast, some regulations were not significant when using these methods. We can suppose, for example, that differences between the models, the prior weights for TF candidates and literature derived pathways, steady state gene expression profiles and corticosteroid drug dynamics in the proposed model may have caused the results. Although some inferred regulations had low significance levels in other approaches, we believe that these regulations can be candidates for true regulation in corticosteroid pharmacogenomic pathways because the proposed method outperformed the other methods through the comparison using synthetic pharmacogenomic pathways.

**Table 5:** The confidence levels of estimated pharmacogenomic regulations using GeneNet and G1DBN.

Regulator	Target	*q*–val	post–prob.
Corticosteroid	Srebf1	0.101	0.000
Corticosteroid	Agtr1a	0.864	0.002
Corticosteroid	Cebpd	0.021	0.003
Corticosteroid	Cebpb	0.747	0.003
Trim63	Serpine1	0.375	0.005
Corticosteroid	Mstn	0.198	0.012
Trim63	Irs1	0.385	0.065
Corticosteroid	Scd	0.905	0.068
Akt2	Mtor	0.881	0.069
Cebpb	Il6r	0.836	0.102
Trim63	Ppard	0.395	0.105
Trim63	Slc2a4	0.915	0.189
Corticosteroid	Ucp3	0.663	0.195
Trim63	Bnip3	0.629	0.217
Trim63	Mstn	0.935	0.273
Mstn	Cebpd	0.413	0.280
Irs1	Ptprf	0.928	0.452
Igf1	Pik3cd	0.897	0.457
Trim63	Mapk14	0.909	0.503
Irs1	Anxa3	0.107	0.632
Irs1	Gpd2	0.853	0.749
Corticosteroid	Edn1	0.833	0.799
Corticosteroid	Pik3c2g	0.929	0.821
Cebpb	Trim63	0.864	0.991
Irs1	Akt3	0.396	1.000
Srebf1	Mapk6	0.453	1.000
Corticosteroid	Scarb1	0.651	1.000
Cebpb	Rxrg	0.734	1.000
Corticosteroid	Ptpn1	0.827	1.000
Srebf1	Irs1	0.832	1.000
Akt2	Cebpb	0.863	1.000
Corticosteroid	Pdk4	0.871	1.000
Cebpd	Pik3c2g	0.888	1.000
Irs1	Foxo1	0.894	1.000
Cebpb	Hmgcs2	0.897	1.000
Corticosteroid	Igf1	0.908	1.000
Trim63	Akt3	0.913	1.000
Cebpd	Igf1	0.924	1.000
Irs1	Bnip3	0.925	1.000
Cebpd	Rheb	0.935	1.000
Trim63	Ace	0.936	1.000

Although we actually used 40 genes, only 35 genes were found to be regulated because the expression of residual genes did not vary through the time-course. Hence the expression of these genes can represent only synthesis and degradation processes, for which regulation was not estimated.

## Discussion

In this study, we proposed a novel method for inference of gene regulatory networks incorporating existing biological knowledge and time-course observation data. The properties of the method are as follows; (i) the dynamics of the gene expression profiles can be estimated based on the proposed linear model with a hidden state, (ii) *L*1 regularized log-likelihood is maximized to infer the active sets of regulation, (iii) the dynamics of other biomolecules can be included in the model, (iv) existing biological knowledge, *e.g.*, literature-recorded pathways and TF information, can be integrated. Furthermore, we proposed an indicator for selecting a simulation time interval for the inference.

To show the effectiveness of the proposed method, we compared it to the previously reported GRNs inference methods using hill function-based pharmacogenomic pathways [38] and a yeast network that is a part of the DREAM4 challenge [39], [40]. Since the artificial simulation models were described by differential equations or difference equations, in which the time intervals were smaller than the measurement interval, to reproduce a realistic biological system, the simulated expressions was updated in detail. In this situation, we assumed that the simulation time interval for the method is crucial for inference. As we expected, the results demonstrated that inference of the regulatory structure depends greatly on the simulation time interval. This indicates that we should carefully design the simulation time interval even for analysis of real observational data. For this purpose, we introduced indicators to determine the simulation time interval and measured their validity. Here, since the tendency of the indicator for the simulation time interval depends on the analyzed biological system, it is recommended to check the tendency by using simulation models. Upon comparison of the inferred structures, the proposed method using the indicator showed the highest performance in terms of precision and recall rates for all three data types. The fact that the proposed method outperformed the other methods in using synthetic datasets, which includes the model we do not assume, indicates the adaptability of our proposed method.

For an application example, we applied the proposed method to a corticosteroid-stimulated pathway in rat skeletal muscle. Because pathways and genes related to corticosteroids have been widely investigated, we were able to obtain the concentration of the drug as a function of time from the corticosteroid kinetics/dynamics and the literature-recorded pathways. By incorporating time-course mRNA expression data, corticosteroid kinetics/dynamics, literature-recorded pathways and TF information, we inferred the regulatory relationships among 40 genes that are candidate or known corticosteroid-related genes. The tendency of the BIC scores and the SPE for the simulated time intervals were the same as in the simulation studies, in which the regulatory systems were based on the previous corticosteroid pharmacogenomic studies, and interesting findings for corticosteroid regulation were obtained. For example, genes that are suggested to be significant factors in corticosteroid pharmacogenomics were predicted to be hub genes regulating other genes in the results both with and without prior information. Furthermore, we found that the properties of the proposed method, *i.e.*, the weighted regularization and inclusion of a term for other biomolecules, influenced the results of selecting potential regulators and introducing drug effects to genes, respectively. Finally, these inferred regulations were evaluated by GeneNet and G1DBN, and some of the regulations had high significance. Since our approach imposed prior weights for reliable regulations and included drug terms to explicitly represent their dynamics, not only these regulations but also regulations that are evaluated as non-significant could be candidate regulations for corticosteroid pharmacogenomics. These results indicate that the proposed method can help to elucidate candidates that will allow extension of GRNs in which the regulation among genes is partly understood by incorporating multi-source biological knowledge.

## Supporting Information

Method S1 **A solution for estimating parameter values and active sets.** The detailed solution of estimating parameter values using the EM-algorithm for VAR-SSM with *L*1 regularization.
(PDF)

Model S1 **Artificial data and parameter values for dataset (i).** The artificial observational data and parameter values for dataset (i).
(ZIP)

Model S2 **Artificial data and simulation files for dataset (ii).** The artificial observational data and a csml file for dataset (ii).
(ZIP)

Model S3 **Artificial data for dataset (iii).** The artificial observational data (100 time-courses) for dataset (iii).
(ZIP)

Model S4 **Corticosteroid pharmacokinetics/dynamics in rat muscle.** A corticosteroid pharmacokinetics/dynamics described in differentia equations in rat muscle [38].
(PDF)
