# Supplementary material for: Inference of Gene Regulatory Networks Incorporating Multi-Source Biological Knowledge via a State Space Model with L1 Regularization
Source: PLoS One. 2014 Aug 27;9(8):e105942. doi: 10.1371/journal.pone.0105942 (PMC4146587; doi:10.1371/journal.pone.0105942)
Supplement: Method S1 — A solution for estimating parameter values and active sets. The detailed solution of estimating parameter values using the EM-algorithm for VAR-SSM with L1 regularization. (PDF) [file pone.0105942.s001.pdf]

Supplement

EM-algorithm for regularized-loglikelihood of VAR-SSM

In the Expectation-step, $q(\boldsymbol{\theta}|\boldsymbol{\theta}_i)$ is calculated by

$$
\begin{aligned}
q(\boldsymbol{\theta}|\boldsymbol{\theta}_i) = &-\frac{1}{2}\log\mid\Sigma_0\mid -\frac{1}{2}\mathrm{tr}\{\Sigma_0^{-1}(\Sigma_{0|T} + (\boldsymbol{x}_{0|T}-\boldsymbol{\mu}_0)(\boldsymbol{x}_{0|T}-\boldsymbol{\mu}_0)')\} \\
&-\frac{T}{2}\log\mid Q\mid -\frac{1}{2}\mathrm{tr}\{Q^{-1}(V_t - V_{lag}F' - FV'_{lag} + FV_{t-1}F' + FE_{t-1}G' + GE'_{t-1}F' - E_{lag}G' - GE_{lag} \\
&+ GZG' + G\boldsymbol{z}\boldsymbol{u}' + \boldsymbol{u}\boldsymbol{z}'G' + F\boldsymbol{s}_{t-1}\boldsymbol{u}' + \boldsymbol{u}\boldsymbol{s}'_{t-1}F' - \boldsymbol{s}_t\boldsymbol{u}' - \boldsymbol{u}\boldsymbol{s}'_t + T\boldsymbol{u}\boldsymbol{u}')\} \\
&-\frac{T}{2}\log\mid R\mid -\frac{1}{2}\mathrm{tr}[R^{-1}\sum_{t=1}^{T}\{(\boldsymbol{y}_t-\boldsymbol{x}_{t|T})(\boldsymbol{y}_t-\boldsymbol{x}_{t|T})' + \Sigma'_{t|T}\}] \\
&-N(T+\frac{1}{2})\log 2\pi - \sum_{n=1}^{N}\sum_{k=1}^{N}\lambda_n|A_{n,k}| - \sum_{n=1}^{N}\sum_{k=1}^{M}\lambda_n|G_{n,k}|, \qquad \text{(S1)}
\end{aligned}
$$

where

$$V_t = \sum_{t\in\mathcal{T}}^{T}(\Sigma_{t|T} + \boldsymbol{x}_{t|T}\boldsymbol{x}'_{t|T}), \qquad \text{(S2)}$$

$$V_{lag} = \sum_{t\in\mathcal{T}}^{T}(\Sigma_{t,t-1|T} + \boldsymbol{x}_{t|T}\boldsymbol{x}'_{t-1|T}), \qquad \text{(S3)}$$

$$V_{t-1} = \sum_{t\in\mathcal{T}}^{T}(\Sigma_{t-1|T} + \boldsymbol{x}_{t-1|T}\boldsymbol{x}'_{t-1|T}), \qquad \text{(S4)}$$

$$\boldsymbol{s}_t = \sum_{t\in\mathcal{T}}^{T}\boldsymbol{x}_{t|T}, \qquad \text{(S5)}$$

$$\boldsymbol{s}_{t-1} = \sum_{t\in\mathcal{T}}^{T}\boldsymbol{x}_{t-1|T}, \qquad \text{(S6)}$$

$$E_{lag} = \sum_{t\in\mathcal{T}}^{T}\boldsymbol{x}_{t|T}\boldsymbol{z}'_{t-1|T}, \qquad \text{(S7)}$$

$$E_{t-1} = \sum_{t\in\mathcal{T}}^{T}\boldsymbol{x}_{t-1|T}\boldsymbol{z}'_{t-1|T}, \qquad \text{(S8)}$$

$$\boldsymbol{z} = \sum_{t\in\mathcal{T}}^{T}\boldsymbol{z}_{t-1|T}, \qquad \text{(S9)}$$

$$Z = \sum_{t\in\mathcal{T}}^{T}\boldsymbol{z}_{t-1|T}\boldsymbol{z}'_{t-1|T}. \qquad \text{(S10)}$$

In the Maximization-step, $\boldsymbol{\theta}_i$ is updated to $\boldsymbol{\theta}_{i+1}$ to be $\boldsymbol{\theta}_{i+1} = \arg\max_{\boldsymbol{\theta}} q(\boldsymbol{\theta}|\boldsymbol{\theta}_i)$. Let $\boldsymbol{v}_{t,n}$, $\boldsymbol{v}_{lag,n}$, $\boldsymbol{v}_{t-1,n}$, $\boldsymbol{e}_{t,n}$ and $\boldsymbol{e}_{lag,n}$ set a transpose of nth row vector of V_t, V_{lag}, V_{t-1}, E_{lag} and E_{t-1}, respectively. Further, set $s_{t,n}$ and $s_{t-1,n}$ as an nth element of $\boldsymbol{s}_t$ and $\boldsymbol{s}_{t-1}$, and $v_{t,n,k}$ and $v_{t-1,n,k}$ as an nth row kth column element of V_t and V_{t-1}, respectively.

Then, $\boldsymbol{\theta}$ is updated as

$$\boldsymbol{a}_n = \arg\min_{\boldsymbol{a}_n} \{\boldsymbol{a}'_n V_{t-1}\boldsymbol{a}_n + 2(1-d_n)\boldsymbol{v}'_{t-1,n}\boldsymbol{a}_n - 2\boldsymbol{v}'_{lag,n}\boldsymbol{a}_n + 2u_n\boldsymbol{s}'_{t-1}\boldsymbol{a}_n + 2\boldsymbol{g}'_n E'_{t-1}\boldsymbol{a}_n + 2q_n\sum_{k=1}^{N}\lambda_n|a_{n,k}|\}, \tag{S11}$$

$$\boldsymbol{g}_n = \arg\min_{\boldsymbol{g}_n} \{\boldsymbol{g}'_n Z\boldsymbol{g}_n + 2\boldsymbol{f}'_n E_{t-1}\boldsymbol{g}_n - 2\boldsymbol{e}'_{lag,n}\boldsymbol{g}_n + 2u_n\boldsymbol{z}'\boldsymbol{g}_n + 2q_n\sum_{k=1}^{M}\lambda_n|g_{n,k}|\}, \tag{S12}$$

$$d_n = 1 - \frac{v_{t,n,n} - u_n s_{t-1,n} - \boldsymbol{v}'_{t-1,n}\boldsymbol{a}_n - \boldsymbol{g}'_n\boldsymbol{e}_{lag,n}}{v_{t-1,n,n}}, \tag{S13}$$

$$\boldsymbol{u} = \frac{\boldsymbol{s}_t - F\boldsymbol{s}_{t-1} - G\boldsymbol{z}}{T}, \tag{S14}$$

$$Q = \frac{1}{T}(V_t - V_{lag}F' - FV'_{lag} + FV_tF' + FE_{lag}G' + GE'_{lag}F' - E_tG' - GE_t + GZG' + G\boldsymbol{z}\boldsymbol{u}' + \boldsymbol{u}\boldsymbol{z}'G' + F\boldsymbol{s}_{t-1}\boldsymbol{u}' + \boldsymbol{u}\boldsymbol{s}'_{t-1}F' - \boldsymbol{s}_t\boldsymbol{u}' - \boldsymbol{u}\boldsymbol{s}'_t + T\boldsymbol{u}\boldsymbol{u}'), \tag{S15}$$

$$R = \frac{1}{T}\sum_{t\in\mathcal{T}_{obs}}^{T}\{(\boldsymbol{y}_t - \boldsymbol{x}_{t|T})(\boldsymbol{y}_t - \boldsymbol{x}_{t|T})' + \Sigma_{t|T}\}, \tag{S16}$$

where d_n is set 0 if $d_n < 0$.

Active sets of parameters of gene regulation

Let $\mathcal{A} = \{\mathcal{A}_1, \dots, \mathcal{A}_N\}$ and $\mathcal{G} = \{\mathcal{G}_1, \dots, \mathcal{G}_N\}$, where $\mathcal{A}_n$ and $\mathcal{G}_n$ be active sets of elements for $\boldsymbol{a}_n$ and $\boldsymbol{g}_n$, *i.e.*, $\forall\{a_{n,k} \neq 0\} \in \mathcal{A}_n$ and $\forall\{g_{n,k} \neq 0\} \in \mathcal{G}_n$ for $k = 1, \dots, N$, respectively. The descriptions of $^{\mathcal{A}_n}$ and $^{\mathcal{G}_n}$ stand for an $|\mathcal{A}_n| \times |\mathcal{A}_n|$ matrix or an $|\mathcal{A}_n|$ dimensional vector and a $|\mathcal{G}_n| \times |\mathcal{G}_n|$ matrix or a $|\mathcal{G}_n|$ dimensional vector, respectively. Then, Eqs. (S11) and (S12) are differentiated to satisfy

$$(S11) \Leftrightarrow \boldsymbol{a}_n^{\mathcal{A}_n} = {V_{t-1}^{\mathcal{A}_n}}^{-1}(\boldsymbol{v}_{lag,n}^{\mathcal{A}_n} - (1-d_n)\boldsymbol{v}_{t-1,n}^{\mathcal{A}_n} - u_n\boldsymbol{s}_{t-1}^{\mathcal{A}_n} - E_{t-1}^{\mathcal{A}_n}\boldsymbol{g}_n^{\mathcal{A}_n} - q_n\lambda_n sign(\boldsymbol{a}_n^{\mathcal{A}_n})), \tag{S17}$$

$$(S12) \Leftrightarrow \boldsymbol{g}_n^{\mathcal{G}_n} = -{Z^{\mathcal{G}_n}}^{-1}(\boldsymbol{e}_{lag,n}^{\mathcal{G}_n} - E_{t-1}^{\mathcal{G}'_n}\boldsymbol{f}_n^{\mathcal{G}_n} - u_n\boldsymbol{z}^{\mathcal{G}_n} - q_n\lambda_n sign(\boldsymbol{g}_n^{\mathcal{G}_n})), \tag{S18}$$

where *sign* means a sign vector consisting positive (+1) or negative (-1) values.
