# Supplementary material for: Inference of Gene Regulatory Networks Incorporating Multi-Source Biological Knowledge via a State Space Model with L1 Regularization
Source: PLoS One. 2014 Aug 27;9(8):e105942. doi: 10.1371/journal.pone.0105942 (PMC4146587; doi:10.1371/journal.pone.0105942)
Supplement: Model S4 — Corticosteroid pharmacokinetics/dynamics in rat muscle. A corticosteroid pharmacokinetics/dynamics described in differentia equations in rat muscle [38]. (PDF) [file pone.0105942.s005.pdf]

Supporting Information Model S4

Corticosteroid kinetics/pharmacodynamics

According to Sun *et al.* (1998), we consider the plasma concentration of corticosteroid as

$$\mathrm{MPL} = C_1 \cdot e^{-\lambda_1 t} + C_2 \cdot e^{-\lambda_2 t}, \tag{SI-1}$$

where C_1 and C_2 are intercepts, λ_1 and λ_2 are the rate of discharges. These values are set as $C_1 = 39,130$ (ng/ml), $C_2 = 12,670$ (ng/ml), $\lambda_1 = 7.54$ (h^{-1}) and $\lambda_2 = 1.20$ (h^{-1}). Using this corticosteroid pharmacokinetic model, Yao *et al.* (2007) established the corticosteroid pharmacodynamic model in rat muscle described by

$$\frac{d\mathrm{mRNA_R}}{dt} = k_{s\_Rm} \cdot \left\{1 - \frac{\mathrm{DR_N}}{\mathrm{IC_{50\_Rm}} + \mathrm{DR_N}}\right\} - k_{d\_Rm} \cdot \mathrm{mRNA_R}, \tag{SI-2}$$

$$\frac{d\mathrm{R}}{dt} = k_{s\_R} \cdot \mathrm{mRNA_R} + R_f \cdot k_{re} \cdot \mathrm{DR_N} - k_{on} \cdot \mathrm{MPL} \cdot \mathrm{R} - k_{d\_R} \cdot \mathrm{R}, \tag{SI-3}$$

$$\frac{d\mathrm{DR}}{dt} = k_{on} \cdot \mathrm{D} \cdot \mathrm{R} - k_T \cdot \mathrm{DR}, \tag{SI-4}$$

$$\frac{d\mathrm{DR_N}}{dt} = k_T \cdot \mathrm{DR} - k_{re} \cdot \mathrm{DR_N}, \tag{SI-5}$$

where $\mathrm{mRNA_R}$ is the concentration of mRNA of the receptor protein, R is the concentration of the receptor protein, DR is the concentration of the drug-receptor complex, $\mathrm{DR_N}$ is the concentration of the drug-receptor complex in nucleus, and *Synthesis* and *Degradation* mean synthesis and degradation processes, respectively. These parameter values are concluded in Table 1. More details can be referred to Yao *et al.* (2007).

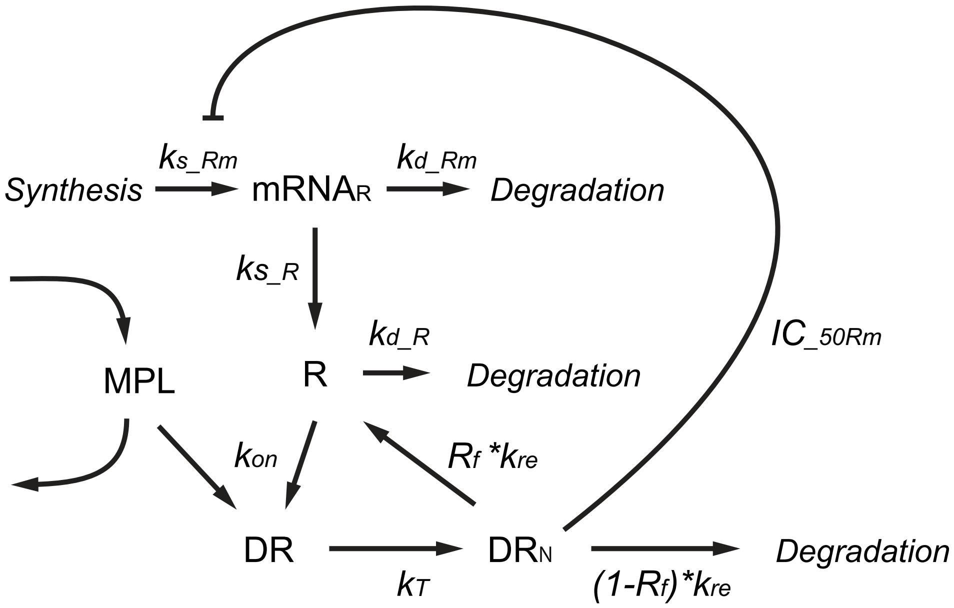

Figure 1: **Corticosteroid pharmacokinetics/dynamics in rat muscle.** This figure illustrates the corticosteroid pharmacodynamics in rat muscle established by Yao *et al.* (2007).

Table 1: **The values of the parameters for corticosteroid pharmacodynamics illustrated in Figure 1.**

parameter	value
$k_{s_R m}$(fmol/g/h)	0.416
$k_{d_R m}$(1/h)	0.139
k_{s_R}(fmol/g/h)	0.777
k_{d_R}(1/h)	0.0356
k_{on}(1/nmol/h)	0.00269
k_T(1/T)	90
k_{re}(1/h)	0.618
R_f	0.720
IC_{50Rm}(fmol/mg)	0.911
GR^0_{mRNA}(fmol/g)	2.99
GR^0(fmol/mg)	65.3
